# A case report of iatrogenic deterioration of yet undiagnosed Rhombencephalitis; always be careful with corticoids

**DOI:** 10.1186/s12907-018-0083-2

**Published:** 2018-12-27

**Authors:** L. Mandigers, J. L. Epker

**Affiliations:** Erasmus Medical Center Rotterdam The Netherlands, Intensive Care Unit – Adults, Rotterdam, The Netherlands

**Keywords:** *Listeria monocytogenes*, Listeria rhombencephalitis, Corticosteroid therapy, Differential diagnosis

## Abstract

**Background:**

*Listeria monocytogenes* is a bacterium present in some food products. It is rarely the cause of Rhombencephalitis in immunocompetent patients.

**Case presentation:**

We report a case of an immunocompetent patient, presenting with progressive perioral numbness and dizziness. Despite treatment with antiplatelet drugs, antiviral medication, antibiotics and corticosteroids for a wide differential diagnosis, the patient deteriorated tremendously. Eventually the patient died of Listeria rhombencephalitis, most likely due to the late diagnosis and concomitant late initiation of antibiotics combined with badly timed and inappropriate corticosteroid prescription.

**Conclusion:**

Early adequate antibiotic treatment is essential in Listeria rhombencephalitis and corticosteroid therapy should be avoided when Listeriosis is suspected.

## Background

Many case reports have been written about the dangers of *Listeria monocytogenes* infection [1–3]. Listeriosis is a food borne disease almost always related to ingestion of non-pasteurized contaminated dairy products or various semi-raw delicacies. Listeria infection is rarely lethal in immunocompetent patients [4]. Several higher risk factors, such as age > 50 years, malignancy and immunocompromised states (chemotherapeutic treatment or corticosteroid therapy) have been described [5]. We report a case of a 67-year old immunocompetent women, who died of undiagnosed Listeria rhombencephalitis, that escalated probably due to the combination of late diagnosis, late adequate antibiotic treatment and inappropriately initiated corticosteroid therapy after 16 days of progressive neurological symptoms.

## Case presentation

**Fig. 1:**
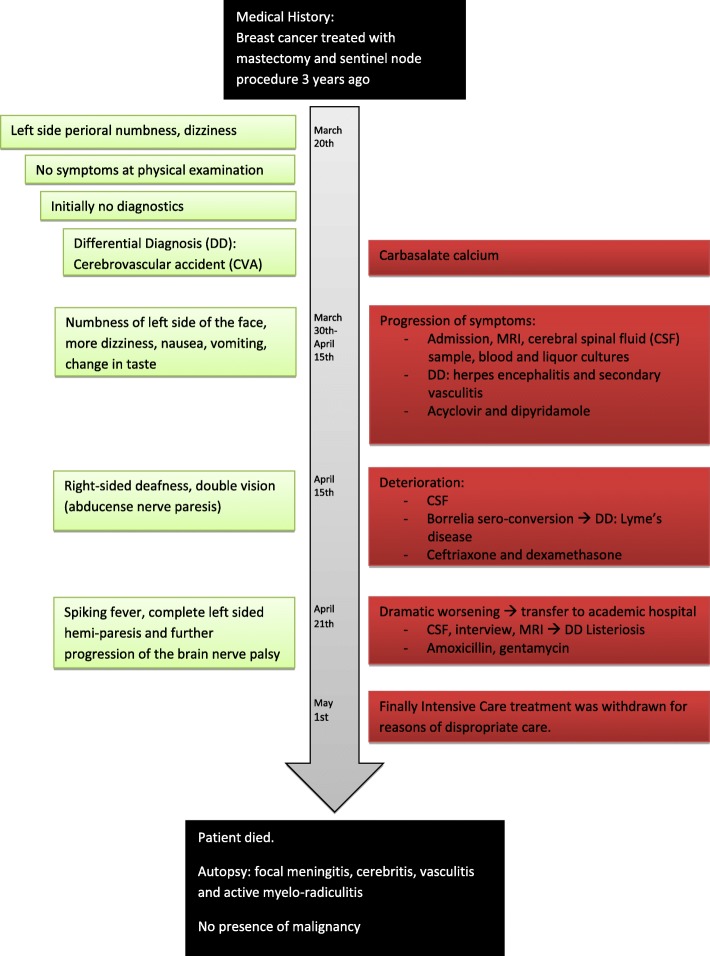
Timetable

A 67-year old woman with a history of mastectomy and sentinel node procedure due to breast cancer three years ago, was seen in an outpatient clinic of a non-academic hospital for progressive perioral numbness and dizziness. At first, a Cerebrovascular accident (CVA) was suspected, so treatment with carbasalate calcium 80 mg once a day was started, see timeline (Fig. 1).

**Fig. 2:**
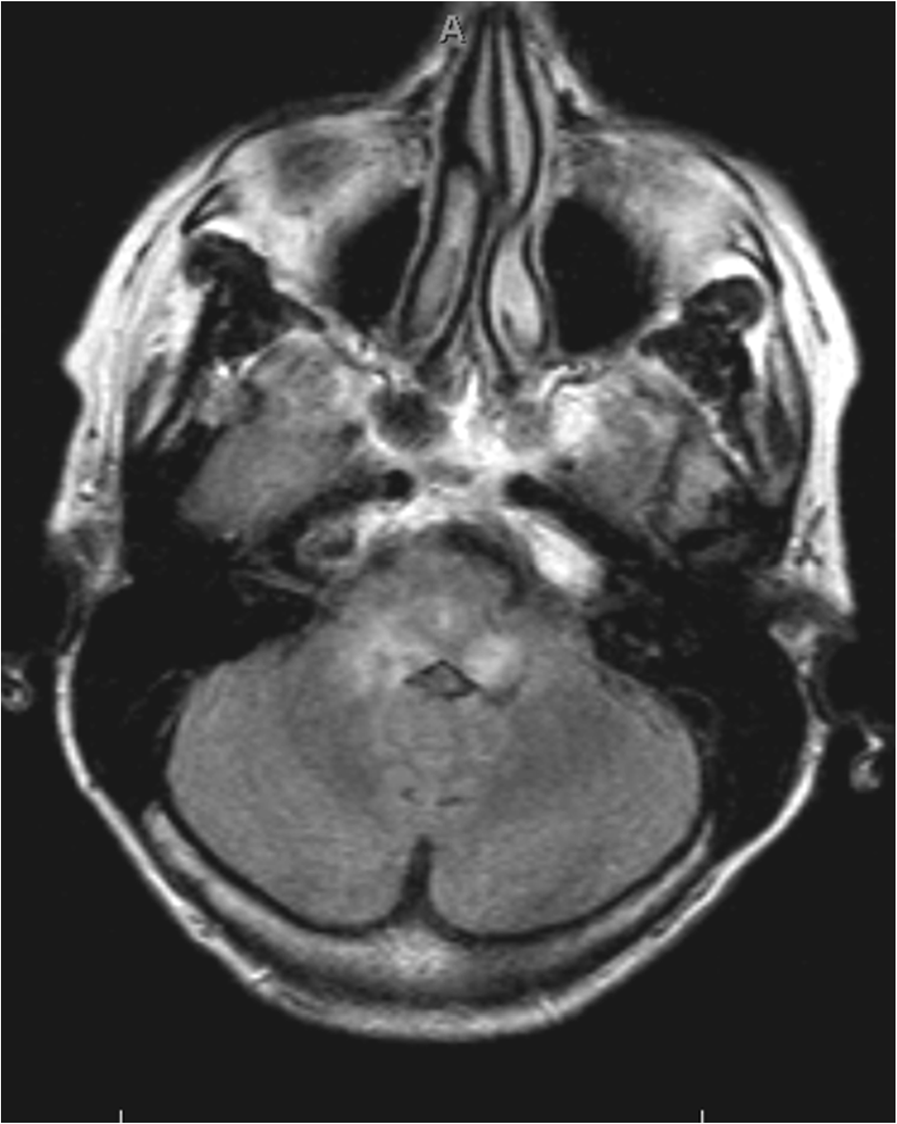
MRI cerebrum Fluid Attenuated Inversion Recovery (FLAIR), diffuse white matter lesions with extension into the pons and medulla oblongata

Despite therapy, the symptoms progressed and the patient was admitted for further analysis. The initial diagnosis of CVA in the vertebrobasilar region was rejected, because the Magnetic Resonance Imaging (MRI) showed fully intact vasculature. However diffuse white matter lesions around the fourth ventricle with extension into the pons and medulla oblongata were seen (Fig. 2).

A cerebral spinal fluid (CSF) sample showed pleocytosis of 240 cells/μL with 30% lymphocytes and a glucose of 3.7 mmol/L. Blood and CSF cultures were negative. With these results, Herpes encephalitis with secondary vasculitis was considered for which acyclovir and dipyridamole was started. A second CSF sample showed pleocytosis of 102 cells/μL with 96% lymphocytes and a glucose concentration of 3.2 mmol/L. Unfortunately, the patient deteriorated, despite the initiated therapy.

At initial presentation the Borrelia serology was negative. However 15 days later the Borrelia IgG became positive with a negative IgM. Because of this unsuspected Borrelia sero-conversion, in combination with a confirmed and recent history of a tick bite, Lyme’s disease was diagnosed. Hence, intravenous therapy with ceftriaxone and dexamethasone was initiated. However after four days, the situation worsened dramatically. The patient developed spiking fever, a complete hemi-paresis and brain nerve palsy progressed. Because of the neurologic deterioration and the diagnostic impasse, the patient was transferred to an academic hospital, three weeks after initial admission.

At presentation in our center, the patient was somnolent (Glasgow Coma Scale (GCS) 14) with a complete left-sided hemiparesis. She had a fever (40.2 °C), high blood pressure (167/70 mmHg), tachycardia (100 beats/min) and tachypnea (25/min). During admission on the neurological ward, the situation rapidly worsened, so transfer to the intensive care unit (ICU) was necessary that same day. On the ICU the patient was intubated because of the risk for aspiration due to a GSC of 7 (E1M4V2), with a concomitant full left sided hemi-paresis. A new CSF sample showed mild pleocytosis of 23 cells/μL and a glucose concentration of 3.4 mmol/L.

**Fig. 3:**
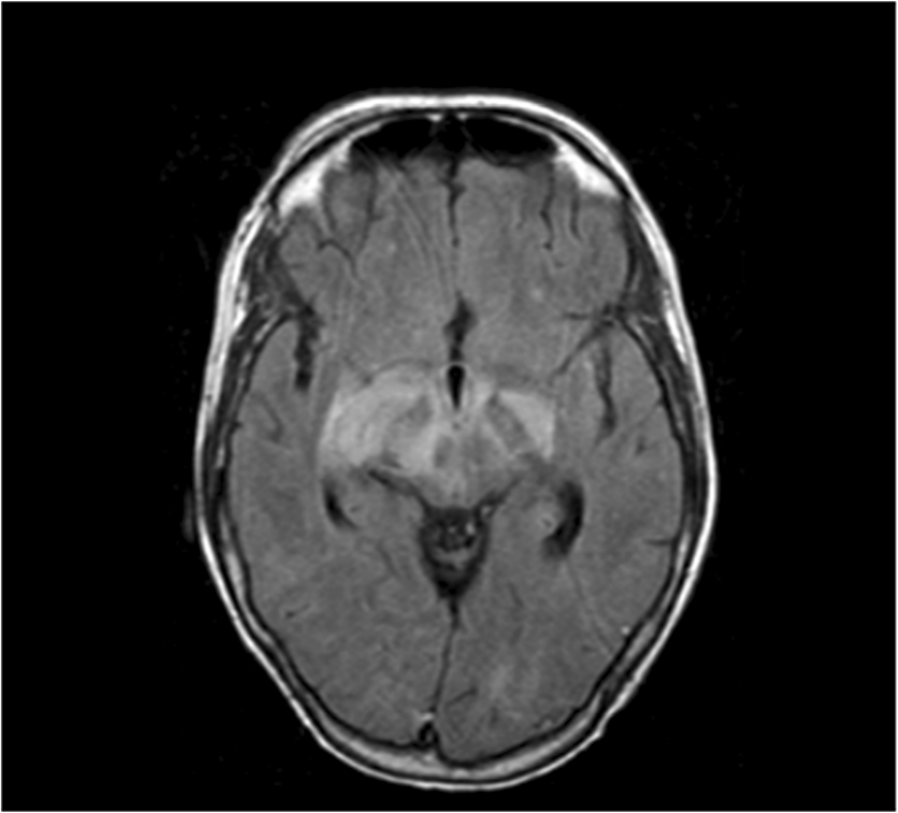
MRI cerebrum (T1), diffuse hyperintensity and swelling, with enhanced ring shaped areas

A new interview with the family directly after ICU admission revealed important and until then unknown information: firstly, the patient appeared to be a professional cat breeder and secondly she was fond of soft French cheeses. This specific and important information had for unexplained reasons not been retrieved until then. Toxoplasmosis had already been ruled out, so Listeriosis appeared to be a likely diagnosis. Intravenous therapy with amoxicillin (2 g, every 4 h) and gentamycin (7 mg/kg once a day) was promptly initiated. Two days later, the blood cultures taken in the non-academic hospital on day 22 of the illness as well as the CSF cultures taken in the academic hospital, showed gram-positive rods, identified as *Listeria monocytogenes*, confirming the diagnosis. A new MRI showed diffuse hyperintensity and swelling of the basal ganglia, with enhanced ring-shaped areas that indicate necrosis (Fig. 3).

Because of further detoriation, persisting GCS 3, absence of brainstem reflexes and in the absence of epileptic activity, intensive care treatment was withdrawn, for reasons of dispropriate care. The patient died a few hours later. Proxy consent for autopsy was obtained.

**Fig. 4:**
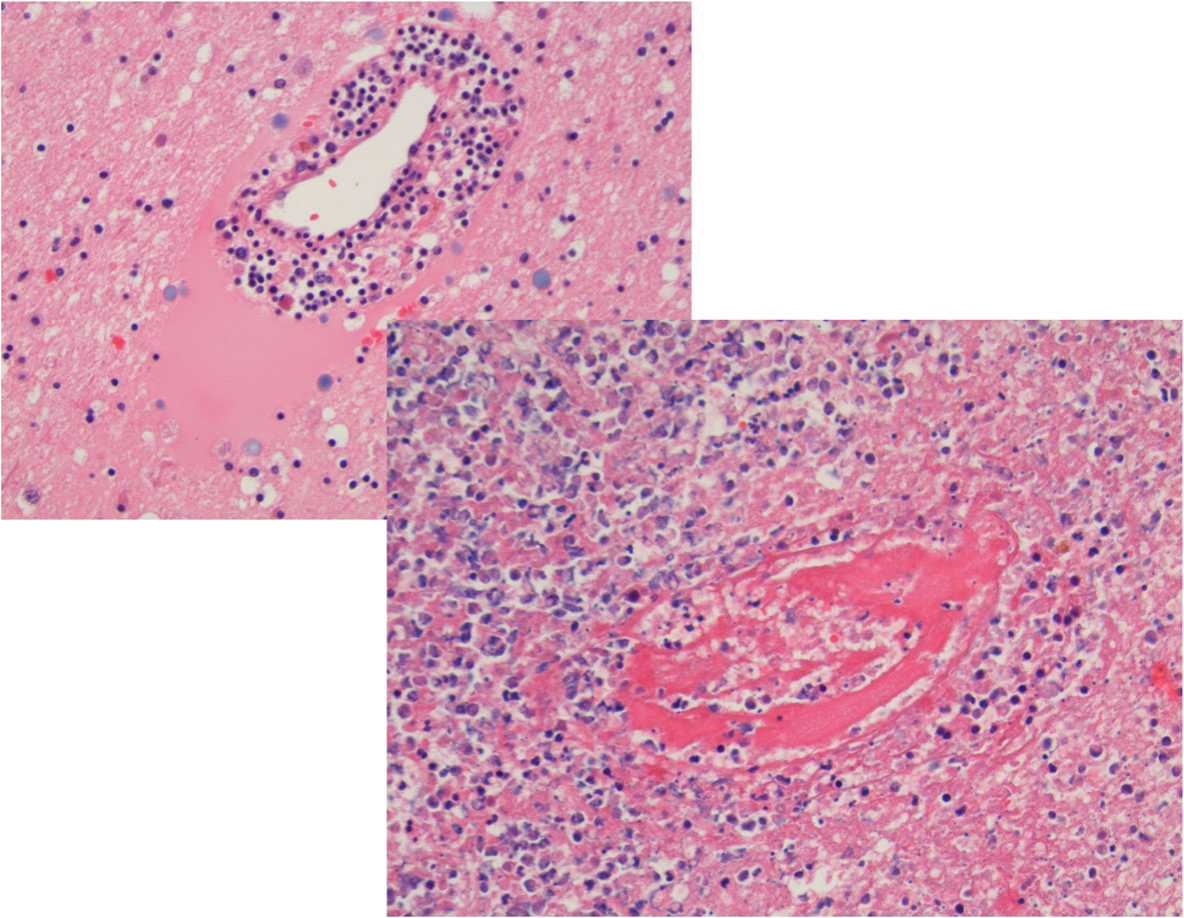
Microscopic findings. Hematoxylin – Eosin coloring, magnification 200x, showing mixed nuclear inflammatory infiltrates as sign of vasculitis of the entire brain with concomitant vascular destruction and trombosis

**Fig. 5:**
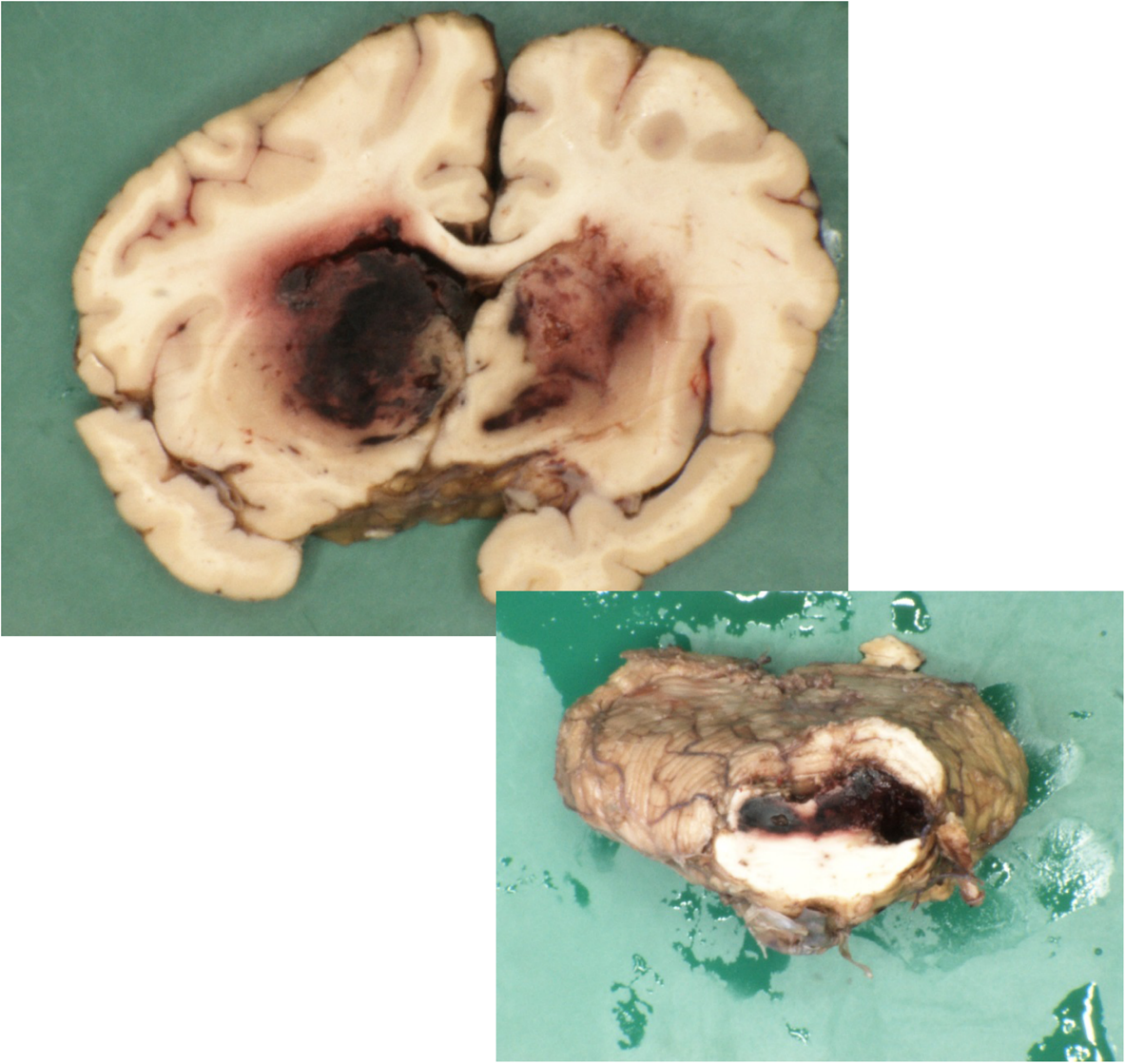
Macroscopic, active vasculitis with diffuse hemorrhagic infarction from the midbrain into the cerebellum

Autopsy revealed no signs of recurrence of breast cancer. There was focal pyogenic meningitis with a mixed nuclear inflammatory infiltrate extending throughout the Virchow Robin spaces, along the vessels, recognized as a myelo-radiculitis. There were signs of vasculitis in almost the entire brain, with complete destruction of the vessels in the basal ganglia (Fig. 4). Also the midbrain showed active vasculitis with diffuse hemorrhagic infarction (Fig. 5). In the right caudate nucleus some active abscesses were still visible.

## Discussion

The clinical presentation of a Listeria infection, after an incubation period ranging from 6 h to up to 90 days, frequently progresses in two phases. In the first phase the patient presents with non-specific symptoms like headache, fever, nausea and vomiting. The second phase includes asymmetric loss of cranial nerve function, ataxia, tremors and other cerebellar symptoms, hemi-paresis, altered consciousness and epileptic fit [4, 6]. Our patient eventually showed almost all of these manifestations, but because of the nonspecific start of the symptoms, *Listeria rhombencephalitis* was only considered in an advanced state. Listeria infection is hard to diagnose because there is no clinical way to separate Listeria infection from many other infectious diseases that can lead to fever, constitutional and/or neurological symptoms. In the future, in case a *Listeria monocytogenes* infection is suspected, a PCR specific for Listeria could be considered [7].

In this case the thorough interview was crucial, because cultures remained negative for a long time. Previous cases also describe the frustrations in the diagnostic process, that are caused by both the atypical presentation and the often negative cultures [2, 3, 8–11]. This emphasizes the need of a structural awareness of the diagnosis of listeria rhombencephalitis and the utmost importance of a structured thorough interview in case of a non-specific clinical presentation. Especially because it can be, although rare, a deathly disease even in immunocompetent patients, as in our patient.

Both Listeria rhombencephalitis and invasive listeriosis have proven to be potential lethal illnesses. The mortality rate differs between 16 and 39% [12, 13]. Rhombencephalitis, in contrary to meningitis, is mostly seen in the immunocompetent patients. Only 8% of the patients presenting with Listeria rhombencephalitis is found to be immunocompromised [6]. In a review by Armstrong and Fung only 8% of the patients used corticosteroids or other immunosuppressive agents and 21% had preexisting medical illnesses [6]. In the Netherlands in the case of bacterial meningitis, corticosteroids are adjunctive on antibiotic treatment, despite the type of organism which is causing the meningitis [14]. A large systematic review by Brouwer et al. showed a non-significant lower mortality rate in corticosteroid treated bacterial meningitis. However they also mention an increase in unfavorable outcome (from 27 to 61%) for Listerial meningitis treated with dexamethasone and suggest to discontinue it when *Listeria monocytogenes* is diagnosed [15]. It should be taken into account that in high-income countries the incidence of Listerial infections is very low. Therefore adding corticosteroid therapy could be justified. In a case described by den Hertog et al., a patient with Rhombencephalitis due to *Listeria monocytogenes* initially responded well at dexamethasone treatment, possibly due to inhibition of inflammatory reaction, without curing the disease [8]. In our case the patient was originally also immunocompetent. Autopsy proved no local recurrence or metastasis of the malignancy of three years ago. However, her condition worsened drastically four days after the initiation of dexamethasone.

Although at last the correct diagnosis was made and the appropriate antibiotics were administered, the patient unfortunately deteriorated and died. This emphasizes that early administration of the correct antibiotic treatment is crucial. In the case of an unknown pathogen, initiation of antibiotics covering the most likely pathogens of the differential diagnosis could be life-saving. Therefore, it leaves no doubt that the marked delay in finding the correct diagnosis eventually has determined the fatal course of events in this case. The administration of corticoids will only have contributed to the final stage of the lethal progression that already had taken its course. The extent of the damage into the entire nervous system in this patient was remarkable. Not only an impressive focal meningitis and cerebritis with concomitant vasculitis and abscesses in the basal ganglia were seen, but also damage to the peripheral nervous system in the form of an active myelo-radiculitis.

## Conclusion

Listerial rhombencephalitis is a challenge to diagnose, especially if cultures remain negative. Therefore a broad differential diagnosis combined with a thorough interview remains essential, because in the end a diagnosis depends merely on what you are looking for. If Listerial infection is not considered or ruled out in infectious patients, late diagnosis and thereby late initiation of antibiotics will allow the disease to run its devastating natural course. The administration of corticosteroids will only catalyze progression of the disease.

## Abbreviations

CSF: Cerebral spinal fluid
CVA: Cerebral vascular accident
GCS: Glasgow Coma Scale
ICU: Intensive care unit
MRI: Magnetic Resonance Imaging
